# Mitochondrial Telomerase Protects Cancer Cells from Nuclear DNA Damage and Apoptosis

**DOI:** 10.1371/journal.pone.0052989

**Published:** 2013-01-09

**Authors:** Chatchawan Singhapol, Deepali Pal, Rafal Czapiewski, Mahendar Porika, Glyn Nelson, Gabriele C. Saretzki

**Affiliations:** 1 Institute for Ageing and Health, Campus for Ageing and Vitality, Newcastle University, Newcastle upon Tyne, United Kingdom; University of Medicine and Dentistry of New Jersey, United States of America

## Abstract

Most cancer cells express high levels of telomerase and proliferate indefinitely. In addition to its telomere maintenance function, telomerase also has a pro-survival function resulting in an increased resistance against DNA damage and decreased apoptosis induction. However, the molecular mechanisms for this protective function remain elusive and it is unclear whether it is connected to telomere maintenance or is rather a non-telomeric function of the telomerase protein, TERT. It was shown recently that the protein subunit of telomerase can shuttle from the nucleus to the mitochondria upon oxidative stress where it protects mitochondrial function and decreases intracellular oxidative stress. Here we show that endogenous telomerase (TERT protein) shuttles from the nucleus into mitochondria upon oxidative stress in cancer cells and analyzed the nuclear exclusion patterns of endogenous telomerase after treatment with hydrogen peroxide in different cell lines. Cell populations excluded TERT from the nucleus upon oxidative stress in a heterogeneous fashion. We found a significant correlation between nuclear localization of telomerase and high DNA damage, while cells which excluded telomerase from the nucleus displayed no or very low DNA damage. We modeled nuclear and mitochondrial telomerase using organelle specific localization vectors and confirmed that mitochondrial localization of telomerase protects the nucleus from inflicted DNA damage and apoptosis while, in contrast, nuclear localization of telomerase correlated with higher amounts of DNA damage and apoptosis. It is known that nuclear DNA damage can be caused by mitochondrially generated reactive oxygen species (ROS). We demonstrate here that mitochondrial localization of telomerase specifically prevents nuclear DNA damage by decreasing levels of mitochondrial ROS. We suggest that this decrease of oxidative stress might be a possible cause for high stress resistance of cancer cells and could be especially important for cancer stem cells.

## Introduction

Telomerase is an enzyme best known for its role in telomere maintenance. Cells with low or no telomerase expression lose telomere repeats during cell division, eventually resulting in cellular senescence. Most cancer cells, germ cells and embryonic stem cells express high levels of telomerase, thus contributing to pluripotency and immortality. In order to maintain telomeres the enzyme needs its catalytic subunit (TERT) as well as the RNA component (TERC or TR) which contains the template for telomere synthesis.

In recent years, however, evidence has accumulated that telomerase, and in particular its catalytic subunit TERT, is involved in various non-telomere-related functions such as regulation of gene expression, growth factors and cell proliferation [1]–[6]. In addition, various groups have shown that TERT shuttles from the nucleus and translocates to mitochondria upon exogenous stress [7]–[12]. We and others have shown a protective role of telomerase within mitochondria [10]–[12] while inability of telomerase shuttling leads to cellular stress, prevents immortalization and increases sensitivity against genotoxic stress, as shown recently by Santos’ group [13]–[15].

Most cancer cells express high levels of telomerase, an important prerequisite for indefinite proliferation and immortality. In addition, telomerase contributes to tumorigenesis via non-telomere dependent mechanisms which are not well understood yet [16].

Telomerase has thus been suggested to be an important anti-cancer target, with the first clinical trials of the telomerase inhibitor imetelstat successfully under way [17], [18]. Telomerase is regulated at multiple levels and subcellular localization is one of them. Cancer cell survival after therapeutic treatments can be heterogeneous with some cells responding to the treatment while others seem to be resistant, contributing to tumor cell survival. A better insight into the biological consequences of different subcellular localizations of TERT might lead to the development of more effective anti-cancer treatments.

Here we characterized the exclusion of telomerase from the nucleus upon stress application and found a heterogeneous stress response in cancer cell populations. Importantly, there was a striking correlation between telomerase/TERT retained within the nucleus and high DNA damage. In contrast, cells which excluded telomerase quickly from the nucleus accumulated no or very low amounts of DNA damage. By modeling the different subcellular localizations of telomerase using organelle-targeted “shooter” vectors we demonstrate here that mitochondrial telomerase prevents nuclear DNA damage as well as the induction of apoptosis after treatment with H_2_O_2_ and irradiation. We suggest that reduced generation of mitochondrial reactive oxygen species (ROS) could be the underlying mechanism to explain how mitochondrial TERT prevents nuclear DNA damage.

Thus, exclusion of telomerase from the nucleus after stress, such as anti-cancer therapeutic treatment could be a protective mechanism that decreases nuclear DNA damage and apoptosis by reducing oxidative stress within mitochondria. This might contribute to increased resistance of those cancer cells against various anti-cancer treatments.

## Results and Discussion

**Figure 1:**
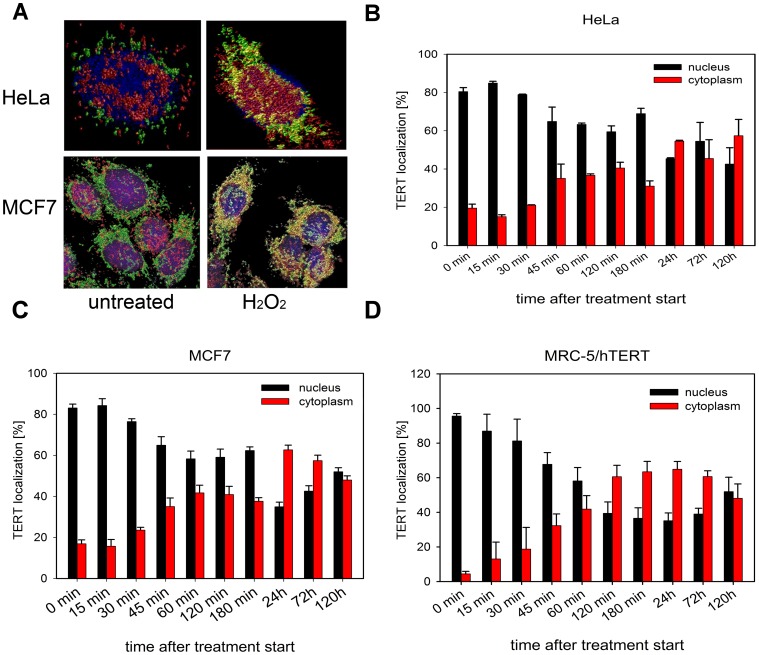
TERT shuttles from nucleus to mitochondria upon H_2_O_2_ treatment in cancer cells. A: Example rendered 3D volume projections of deconvolved confocal images from HeLa and MCF7 cells untreated (control, left panel) or treated with 400 µM H_2_O_2_ for 3 h (right panel). Green represents mitotracker green fluorescence, red anti-TERT immuno-fluorescence and blue nuclear DNA (DAPI). Marked colocalization between mitotracker green and TERT is displayed by red-green mixing being displayed as yellow. **B–D:** TERT localization kinetics in 3 cell line populations after treatment with 400 µM H_2_O_2_ over 5 days. **B:** HeLa **C:** MCF7 **D:** MRC-5/hTERT. Black bars: nuclear TERT, red bars: cytoplasmic TERT. Bars are means ± SE from at least 30 cells per time point and cell line from 3 independent experiments.

Subcellular shuttling of TERT protein from the nucleus to mitochondria had been shown previously in various cell types, including cancer cells [7], [10]–[12]. We confirmed this shuttling of endogenous telomerase after H_2_O_2_ treatment in HeLa and MCF7 cells (Fig. 1A) from the nucleus to mitochondria and quantified the exclusion compared to MRC-5/hTERT cells (Table 1). In order to evaluate the shuttling kinetics of TERT in more detail we analyzed 3 cell lines, including 2 cancer cell lines as well as hTERT over-expressing MRC-5 fibroblasts, and followed them over 5 days.

**Table 1:** Quantification and significances of z-stacks for determination of correlation coefficient for co-localization of hTERT to mitochondria in three cell lines.

Cell type	H_2_O_2_ treatment	Correlation coefficient for co-localization of hTERT to mitochondria	P-value
MRC5/hTERT	−	0.702	
MRC5/hTERT	+	0.891	**0.0047**
HeLa	−	0.579	
HeLa	+	0.887	**0.0180**
MCF7	−	0.475	
MCF7	+	0.795	**0.0182**

Mean correlation coefficients were determined from between 8–10 cells per cell line/treatment. Pearson correlation coefficients were determined per cell for deconvolved, 3D rendered images, subtracting any background for each channel, using Huygens Colocalization analyzer plugin (Huygens, SVI, Netherlands). All datasets passed Normality tests and P values are from Student's T tests comparing untreated and treated cells per cell line.

In all 3 cell lines nuclear TERT exclusion started around 45 min after onset of H_2_O_2_ treatment. In hTERT over-expressing fibroblasts the exclusion reached its maximum of 60% after 3 hours while it took both cancer cell lines up to a day to reach an exclusion level of 50–60% (Fig. 1B–D). This corresponds well with the TERT mitochondrial co-localization data from our confocal images from the 3 cell lines (Table 1). Intriguingly, the nuclear exclusion level of 50–60% persisted in all 3 cell lines up to 5 days, the longest time points we analyzed (Fig. 1B–D and S1). Thus, nuclear TERT exclusion is a rather persistent process that can last up to several days after a single bolus dose of 400 µM H_2_O_2._ To our knowledge, this is the first time that the TERT exclusion kinetics has been investigated in detail and compared in three different cell lines over a 5 day time frame. The long persistence of TERT protein outside the nucleus in the cancer cell lines might be an important contributor to increased resistance and decreased apoptosis in cancer cells after drug treatment or irradiation compared to non-cancer cells which in most cases express no or rather low levels of telomerase. We have shown previously that telomerase negative fibroblasts are much more susceptible to apoptosis after treatment with H_2_O_2_ and etoposide than their telomerase over-expressing counterparts [10]. We had also shown previously [10] that TERT exclusion from the nucleus is reversible over a time frame of around 10 days. However, we do not know whether the persistent TERT protein within mitochondria does always come from the nucleus or whether newly synthesized TERT protein is directly imported into mitochondria. This question requires further investigation.

Next we correlated telomerase exclusion for each individual cell with its DNA damage level at 3 hours after H_2_O_2_ treatment. We quantified the TERT exclusion level for each single cell as either nuclear, if more than 75% of total TERT was in the nucleus, or cytoplasmic/mitochondrial if more than 75% of TERT was outside the nucleus with the remaining cells being classified as an intermediate phenotype.

**Figure 2:**
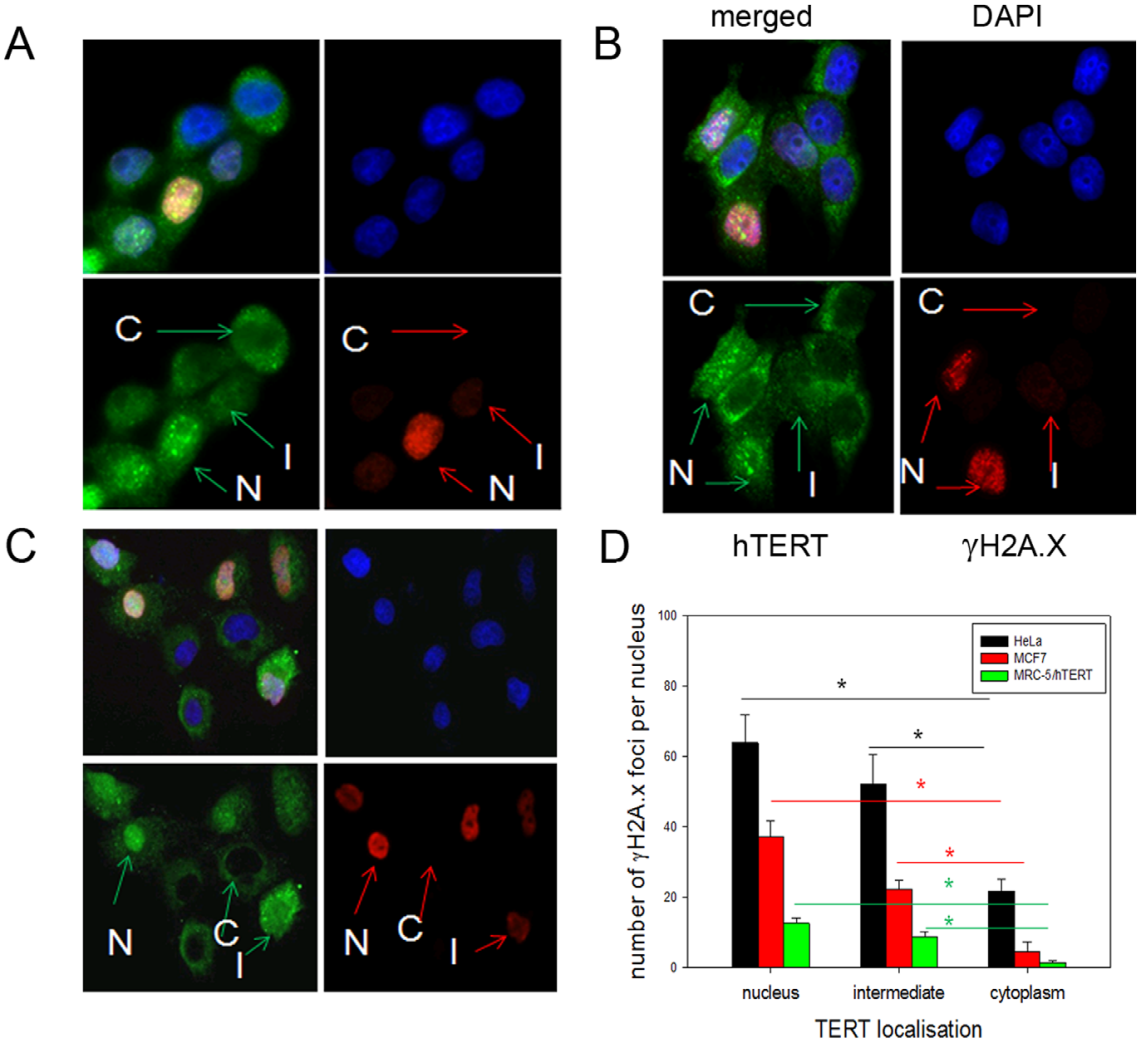
Nuclear TERT localization correlates with high DNA damage levels after treatment with H_2_O_2_ **while mitochondrial telomerase prevents it. A–C:** Representative images of TERT localization (green), and γH2A.X staining (red). Blue: DAPI nuclear counterstain **A:** HeLa **B:** MCF7 **C:** MRC-5/hTERT cells. Cells were treated for 3 h with 400 µM H_2_O_2_. TERT localization was determined as described for Figure 1B and grouped into 3 categories: nuclear TERT (N) TERT (C) and intermediary TERT (I) localization. Examples for the 3 different localizations are indicated with arrows. **D:** Correlation between subcellular TERT localization and nuclear DNA damage levels (number of γH2A.X foci). Cytoplasmic TERT localization correlates with low nuclear DNA damage in all 3 cell lines while nuclear TERT localization results in high nuclear damage after 3 h of treatment with 400 µM H_2_O_2_. Intermediary TERT localization results in intermediate DNA damage levels. Black bars: HeLa, red bars: MCF7, green bars: MRC-5/hTERT. Bars are mean ± SE from at least 40–100 cells per cell line in repeated experiments. * P<0.05.

We found a clear heterogeneity for nuclear TERT exclusion between cells within a population (Fig. 2A and S2A). Intriguingly, we found that cells that had excluded telomerase 3 hours after the treatment showed no or very low DNA damage whilst those with nuclear telomerase had a significantly higher amount of nuclear DNA damage in all 3 cell lines (Fig. 2). Also, cells with an intermediate exclusion pattern showed an intermediate damage level, still significantly higher than cells with completely excluded telomerase but not significantly different from those with predominantly nuclear telomerase. This data suggests that a high level of nuclear exclusion (>75%) and mitochondrial localization of TERT is required in order to exert its protective function [10]–[15]. Absolute DNA damage levels were different between the 3 cell lines with the two cancer cell lines displaying much higher damage levels than TERT over-expressing fibroblasts. We also measured the absolute TERT signal intensities for the 3 different localizations and found that mitochondrial TERT signal was always lower than that in the nucleus or in the intermediate state (Fig. S2B). It had been shown previously that TERT protein level is down regulated within mitochondria after H_2_O_2_ treatment which could explain this observation [19].

A correlation between higher DNA damage in cancer cells and nuclearly confined telomerase unable to shuttle due to a mutation in its nuclear export signal was described recently by Kovalenko and colleagues [13]. The mutated TERT induced an increase in spontaneous telomeric as well as non-telomeric nuclear DNA damage in 2 cancer cell lines compared to the same cells without the mutant TERT [13]. In addition, cancer cells with a mutant TERT that was confined to the nucleus and unable to shuttle lost their proliferation ability, were not able to form colonies in soft agar and showed an increased amount of mitochondrial DNA damage [13]. Together, these results suggest that sub-cellular shuttling of TERT might have important implications for the sensitivity of cells against DNA damage. This increased resistance due to high telomerase expression and nuclear exclusion of TERT might favor the survival of cancer stem cells which could result in relapse after therapy [17]. There is also previously published data of a protective role of nuclear TERT against staurosporine induced apoptosis [9]. However, staurosporine is a protein kinase inhibitor that activates apoptosis in a rapid manner without inducing DNA damage and independent from mitochondria. We therefore suggest a different mechanism of action in both experiments.

**Figure 3:**
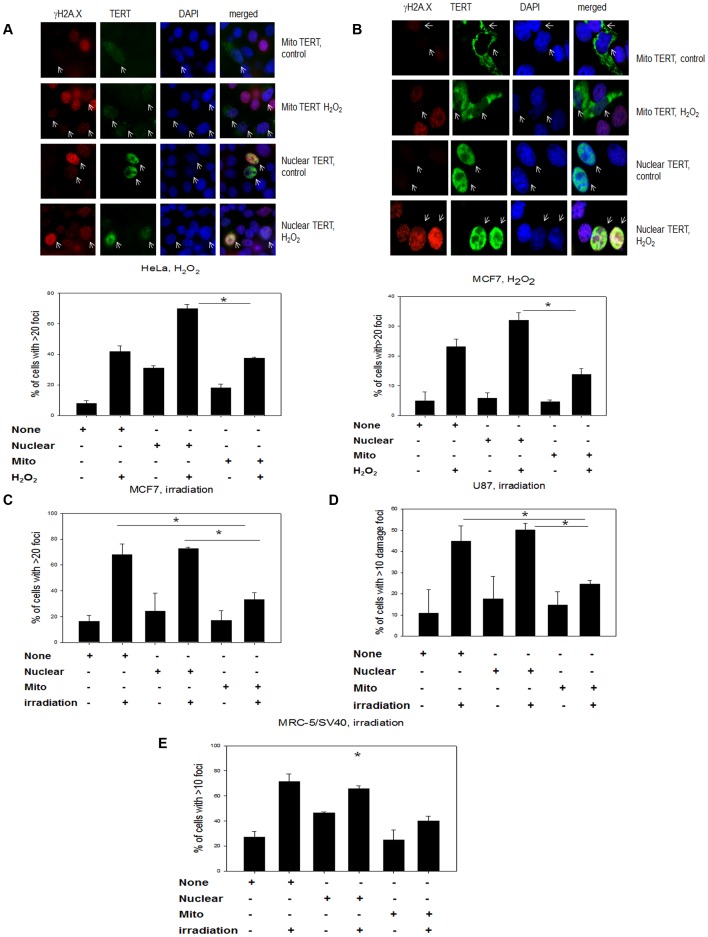
Mitochondrially located TERT reduces nuclear DNA damage after H_2_O_2_ **treatment in comparison to nuclear TERT localization in 4 different cell lines. A**: Organelle specific TERT vectors transfected into HeLa cells. Upper panel: representative images of cells transfected with mitochondrial and nuclear TERT shooter vectors with and without treatment with 200 µM H_2_O_2_ for 3 hours. TERT staining (using myc-tag) fused to TERT protein (green) and γH2A.X staining (red) for DNA damage foci. Arrows show transfected cells. Lower panel: Quantification of cells with high levels of DNA damage foci for transfected and un-transfected cells with and without H_2_O_2_ treatment. Bars are mean ± SE from 3 independent experiments, *P<0.05. **B**: Organelle specific TERT vectors transfected into MCF7 cells. Panels as described for A. **C–F**: Quantification of cells with high levels of DNA damage foci for transfected and un-transfected cells with and without x-irradiation. **C**: MCF7 after 20 Gy X- irradiation. **D:** U87 after 20 Gy X-irradiation. **E**: MRC-5/SV40 after 10 Gy X-irradiation. Bars are mean ± SE from 3 independent experiments. * P<0.05.

We next modeled the different TERT localizations separately by over-expressing nuclear and mitochondrial organelle specific vectors expressing the catalytic telomerase subunit TERT fused to a myc-tag in 3 cancer cell lines: HeLa, MCF7 and U87 glioblastoma (Fig. 3). Transiently transfected cells were treated either with H_2_O_2_ or irradiation and analyzed for γH2A.X DNA damage foci and TERT localization using the fused myc-tag. The localization for mitochondrial (mito TERT) and nuclear TERT is shown in Fig. 3A and B (upper panels). There was no difference in DNA damage levels between cells transfected with either vector or un-transfected cells before treatment. However, we found that in all 3 cell lines and both treatments, cells with a mitochondrial TERT localization had significantly less DNA damage compared to those cells that either expressed nuclear TERT or were un-transfected (Fig. 3 A–D). In order to exclude that endogenous telomerase interacted with the over-expressed shooter TERT protein we repeated the experiment in an SV40 immortalized MRC-5 cell line that maintains its telomeres via an alternative lengthening mechanism [20]. Post irradiation, we found the same protective effect of mitochondrial telomerase (Fig. 3 E). We also used an antibody against 53BP1, another protein involved in the DNA damage response to confirm our results that DNA damage foci are high in cells transfected with nuclear TERT while in contrast those transfected with mitochondrial TERT show less DNA damage than un-transfected cells (Fig. S3).

**Figure 4:**
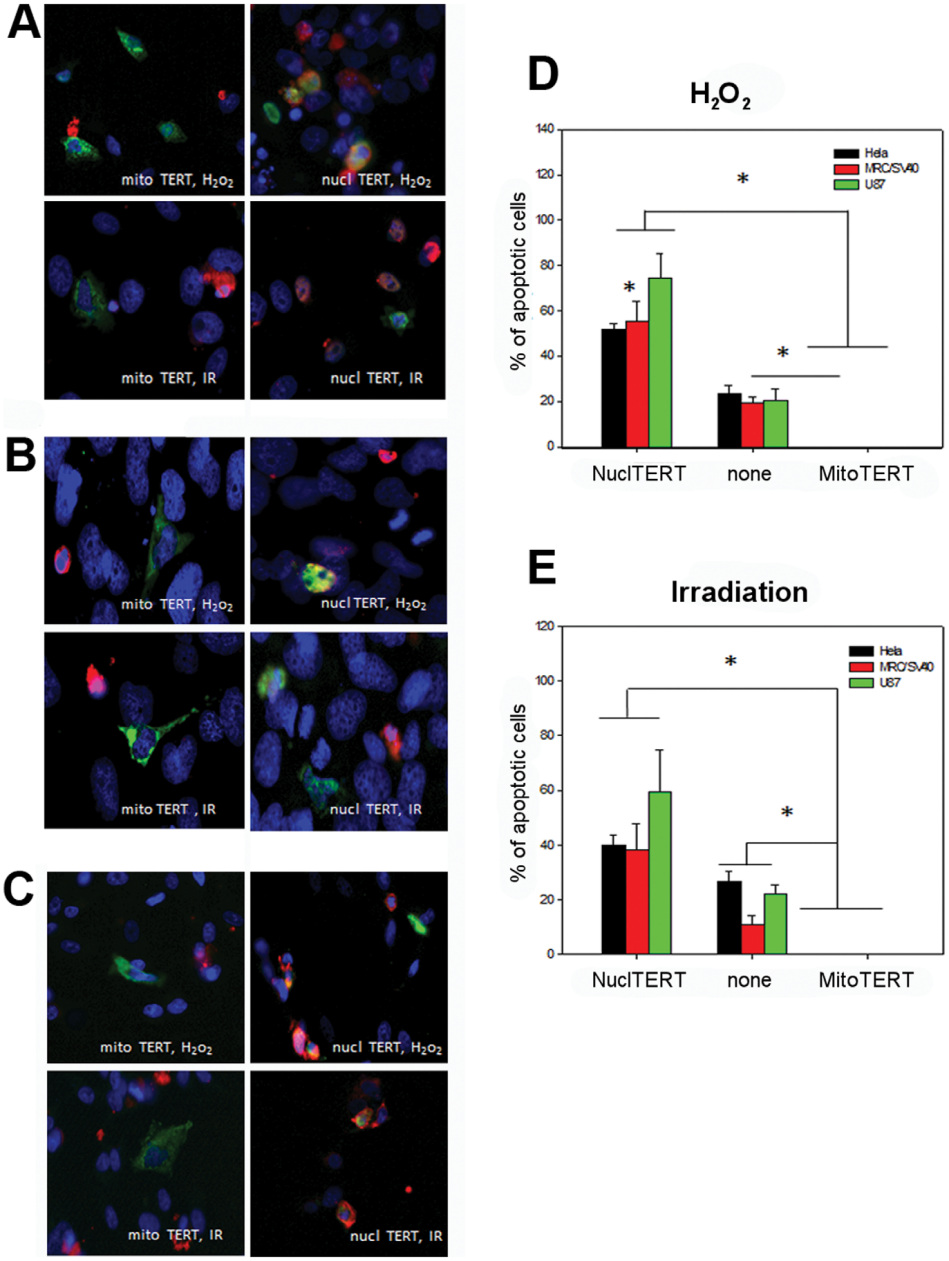
Mitochondrial TERT protects from apoptosis induction after H_2_O_2_ treatment and X-irradiation compared to cells transfected with nuclear TERT. Representative images of activated caspase 3 (shown in red) in **A**: Hela, **B**: MRC/SV40, **C**: U87 cells transfected with mito TERT and nuclear TERT (myc-tag, shown in green) after 400 µM H_2_O_2_ treatment for 3 h or irradiation with 20 Gy. **D**: Quantification of the percentage of apoptotic cells of the 3 cell lines after H_2_O_2_ treatment, E: Quantification of the percentage of apoptotic cells of the 3 cell lines after X-irradiation. Bars present mean and standard error from around 45 transfected cells per condition and cell line. * p<0.05.

**Figure 5:**
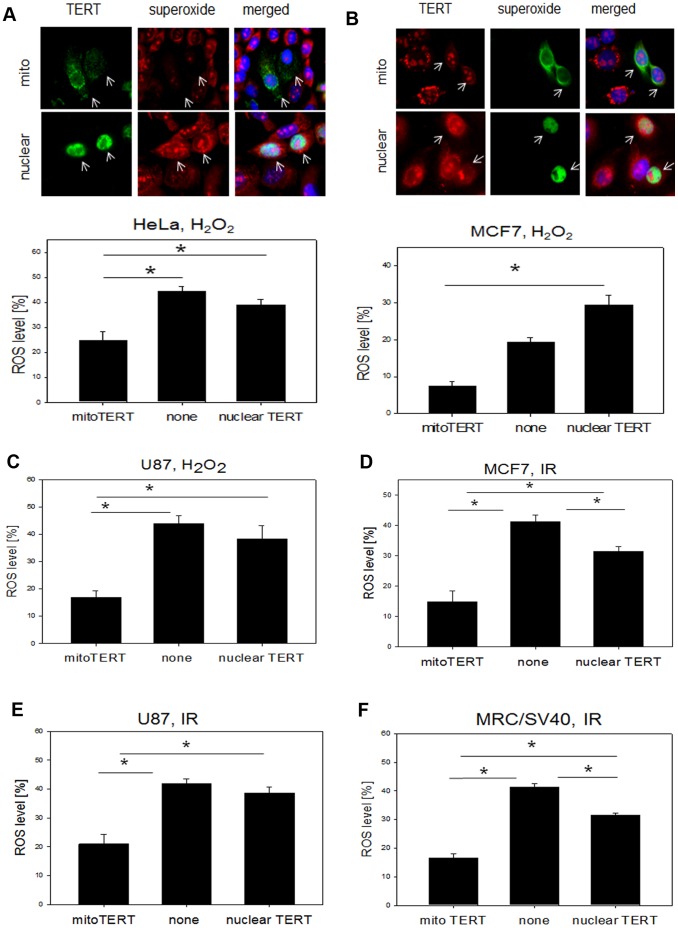
Mitochondrially localized TERT protects against mitochondrial ROS generation after H_2_O_2_ treatment and irradiation in 4 different cell lines. A: Upper panel: Representative images of ROS staining (red, mitosox) and TERT localization (myc-tag, green) after organelle specific TERT transfection and 100 µM H_2_O_2_ treatment for 3 h in HeLa cells. Upper row: mito- TERT, lower row: nuclear TERT. Arrows indicate transfected cells. Lower panel: Quantification of ROS levels measured as percentage of mitosox positive area from whole cytoplasm using ImageJ in transfected and un-transfected cells. **B:** MCF7 cells, panels as described for A. **C:** Quantification of ROS in U87 cells after 3 h of 100 µM H_2_O_2_ treatment. **D–F:** Quantification of ROS levels after X-irradiation. **D:** MCF7 after 20 Gy X-irradiation. **E:** U87 after 20 Gy X-irradiation **F:** MRC-5/SV40 after 10 Gy X-irradiation. Bars represent mean ± SE from 3 independent experiments. * P<0.05.

Since high amounts of nuclear DNA damage are thought to decrease survival of cells we analyzed whether the used stress treatments would also compromise cell survival and induce apoptosis. We treated 3 cell lines transfected with both TERT shooter vectors with H_2_O_2_ and X-irradiation and determined apoptosis induction using an antibody against activated caspase 3. Intriguingly, not a single cell transfected with mitochondrial TERT showed any sign of apoptosis while around 20% of un-transfected cells and between 40–60% of cells expressing the nuclear shooter were apoptotic (Fig. 4). This result confirms that indeed the induced DNA damage found in cells with nuclear TERT localization impacts directly on cell survival while mitochondrial TERT efficiently protects against apoptosis. In order to elucidate the mechanism by which mitochondrial TERT might protect cancer cells from nuclear DNA damage we measured the amount of mitochondrial ROS after H_2_O_2_ treatment and irradiation using mitosox staining as a measure of mitochondrial superoxide generation in addition to myc-TERT staining for nuclear and mitochondrial “shooter” vectors in the same 3 cancer cell lines (Fig. 5 A–E). Mitosox dye is taken up by mitochondria in a membrane potential specific manner. In order to exclude that different membrane potential caused the effects, we measured mitochondrial membrane potential in HeLa and MCF7 cells transfected with both TERT shooters and compared them to un-transfected cells after H_2_O_2_ treatment as well as X-irradiation. In accordance with our previous findings of an increased mitochondrial membrane potential in MRC-5/hTERT compared to parental fibroblasts the results confirm a significantly higher membrane potential in cells over-expressing mitochondrial TERT, which is in HeLa cells already apparent even before any stress treatment (Fig. S4). Therefore, mitosox levels found in our experiments truly represent different ROS levels which are dependent on TERT localization. We found that over-expression of mitochondrial TERT in all cell types resulted in significantly lower ROS levels after H_2_O_2_ treatment and irradiation compared to un-transfected cells or those that over-expressed nuclear TERT (Fig. S4).

Again we used MRC-5/SV40 cells without endogenous telomerase to confirm the results from the 3 cancer cell lines and found the same protective effect of mitochondrially localized TERT on ROS levels (Fig. 5F). ROS levels in cells expressing the nuclear TERT “shooter” were usually not different from un-transfected cells with the exception of MCF7 and MRC-5/SV40 cells after irradiation, where nuclear shooter transfected cells showed lower ROS levels than un-transfected cells.

This data suggests that by shuttling into mitochondria telomerase/TERT not only protects the organelle, but by decreasing mitochondrial superoxide production also indirectly protects the nucleus from DNA damage. Similar observations of telomerase shuttling from the nucleoplasm to nucleoli had been reported previously under ionizing radiation in primary and cancer cells [21]. The authors had speculated that telomerase might negatively interfere with repair enzymes in the nucleus under conditions of increased DNA damage and stress. Our results seem to support this suggestion that telomerase might be “undesirable” within the nucleus under conditions of DNA damage. Telomerase has been shown to “heal” chromosomes by attempting a form of DNA repair by adding telomere sequences to broken telomere ends [22]. However, this might not lead to proper DNA repair, as known to be carried out by true DNA repair systems.

Our results demonstrate that mitochondrial telomerase localization specifically decreases mitochondrial ROS generation and cellular oxidative stress after induction of exogenous stress generated by H_2_O_2_ or irradiation in cancer cells and might thereby prevent damage to nuclear DNA. It could also explain why shuttling of telomerase from the nucleus to mitochondria seems to promote cellular survival, whilst in cells where telomerase is not able to leave the nucleus DNA damage accumulation is observed.

Diehn and colleagues reported recently that cancer stem cells that produced less ROS due to higher antioxidant expression accumulated less DNA damage after ionizing radiation [23]. It would be interesting to ascertain whether these cells also have more excluded telomerase than non-cancer stem cells after irradiation. It has been shown that cancer stem cells express high telomerase activity level; however nothing is known about TERT shuttling in these cells [24].

We have demonstrated previously that exogenous ROS generation by irradiation in fibroblasts damages mitochondria and accelerates nuclear DNA damage creating a positive feedback loop [25]. We suggest that such a functional interaction between mitochondria and the nucleus also exists in cancer cells and other telomerase positive cells, where telomerase enters mitochondria in order to decrease ROS which are induced by chemotherapeutic drugs and irradiation. Thus, it seems that anti-cancer treatments can induce a novel, hitherto unknown mechanism of telomerase shuttling that prevents nuclear DNA damage by decreasing mitochondrial ROS generation via induction of telomerase shuttling. Due to its heterogeneous pattern it could also explain the resistance of some, but not all, cancer cells against therapeutic treatments.

## Materials and Methods

### Cells

HeLa, MCF7, U87 and MRC5/SV40 cells originated from ATCC. MRC5 were purchased from ECACC (London). hTERT overexpression was performed using retroviral transfection of hTERT as described earlier [10]. U 87 and MRC5/SV40 cells were cultivated in MEM and DMEM respectively supplemented with 1% non-essential amino acid, 10% FCS (Sigma), 2 mM glutamine, and 1% penicillin/streptomycin. All other cell types were cultivated in DMEM (PAA) containing 10% FCS (SIGMA), 1% penicillin/streptomycin (PAA) and 2 mM glutamate (Gibco). Cells were incubated at 37°C at ambient oxygen and 5% CO_2_.

### Treatments

Cells were seeded 1 or 2 days before treatment onto 19 mm coverslips in 12 well plates for immuno-fluorescence staining (5×10^4^ per well).

H_2_O_2_ (SIGMA) treatment was performed in serum free medium for the indicated times and concentrations. The used H_2_O_2_ concentrations had been optimized for each different type of experiment. X-irradiation at 10 and 20 Gy was performed using a Faxitron (Elektron Technology, UK). For all irradiation experiments (except apoptosis analysis) cells were fixed and analyzed 20 minutes after treatment. Cells were fixed using 4% paraformaldehyde in PBS for 10 min.

### Immuno-fluorescence Staining and Imaging

Cells were fixed on coverslips using 4% paraformaldehyde for 10 min. Single or double immuno-staining was performed with the following primary antibodies: mouse γH2A.X (Upstate), rabbit anti-TERT (Rockland), rabbit anti-Ki67 (Abcam) and anti myc-tag (Abcam). Specificity and lack of background staining of the used TERT antibody has been confirmed (Fig. S5). Secondary antibodies were: goat anti-mouse and rabbit Alexafluor 594 and 488 (Molecular probes/Invitrogen). Nuclear staining was observed using DAPI. Images for each channel were obtained using an AxioImager Z1 microscope (Zeiss) equipped with suitable filter cubes to spectrally distinguish Alexafluor^488^ and Alexafluor^594^, ensuring no bleedthrough into/from either fluorophore (established previously, data not shown). Images were subsequently analysed using ImageJ. Thresholds were defined for each image individually.

### Confocal Fluorescence Microscopy and Co-localization Analysis

Cells were loaded with 400 nM mitotracker green (Molecular probes) for 30 minutes at 37°C before fixation and then stained with anti-TERT antibody and Alexa Fluor® 594. Images were captured using a Zeiss LSM 510 equipped with a 63×1.4 NA objective with the pinhole set to 1 Airy unit (Zeiss, Germany). Z stacks were obtained every 100 nm for each cell (sampling >2×Nyquist criteria). Images were deconvolved and then analyzed for object co-localization in 3D using Huygens software (SVI, Netherlands). For colocalization analysis and image preparation, the deconvolved images were rendered into 3 dimensional volumes. Single cells were isolated within the image and single, unified objects (mitochondrial and Tert) were identified using 'Object Colocalization' in Huygens with a cut-off to exclude any objects smaller than one mitochondrion in the mitotracker green channel. Huygens then computed a Pearson's correlation coefficent for each cell for 3 dimensional colocalization between mitochondria and Tert staining. This enabled us to accurately predict true colocalization (within the limits of diffraction limitation) of the two dyes irrespective of their different wavelengths.

### Organelle Specific Transfection

TERT containing nuclear and mitochondrial organelle specific vectors (pCMV-myc-mitoTERT and pCMV-myc-nucTERT were a kind gift from J. Haendeler and Joachim Altschmied, Duesseldorf, Germany and described previously [9], [11]). Transient transfection was performed using lipofectamine ™ 2000 (Invitrogen, USA) of mito-TERT and nuc-hTERT “shooter” vectors. The average transfection efficiencies 48 hours after transfection were between 25 and 30%. Transiently transfected cells were treated 2 days after transfection either with H_2_O_2_ or irradiation.

### Determination of ROS Level

Cells were stained with 5 µM mitosox (Invitrogen, USA) for 15 min after H_2_O_2_ treatment or irradiation prior to fixation and antibody staining. ROS levels were determined as the percentage of cytoplasmic mitosox signal from total cytoplasmic area using Image J (http://rsbweb.nih.gov/ij/).

### Analysis of DNA Damage

Analysis of DNA damage was performed using immuno-fluorescence either as a single staining with γ-H2A.X or double staining with TERT. Cells were fixed, permeabilized with PBG (PBS, BSA, fishskin gelatine and 0.5% triton) and γ-H2A.X antibody was applied to the cells and then stained with Alexa Fluor® 594. Anti myc-tag and Alexa Fluor® 488 were used to visualise transfected TERT protein. Slides were examined using a Zeiss Axiovision fluorescence microscope (Zeiss, Germany). For the analysis of DNA damage the number of DNA damage foci was counted for each type of TERT localization separately from 20–40 cells per group and cell line.

### Measurement of TERT Exclusion Rate

For each individual cell, TERT localization was manually quantified by comparing telomerase signals inside and outside the nucleus using Image J. Subcellular areas were determined for nuclear and cytosolic regions by using freehand selection. Expression signals in the selected area were evaluated using area calculation function after thresholding to remove noise. The result of each individual cell indicated a percentage of TERT signal expressed in the subcellular compartment:

% TERT nuclear expression = TERT signal in nucleus area/total TERT signal×100,

% TERT cytoplasmic expression = TERT signal in cytosolic area/total TERT signal×100. The average percentage of nuclear and cytoplasmic localization of TERT from at least 30 individual cells was taken to determine the average percentage of the whole population.

### Correlation of Cellular TERT Localization and DNA Damage Level

We classified the localization of TERT into 3 classes: nuclear TERT (N): 75%–100% of TERT signal resides within the nucleus, cytoplasmic TERT (C): 75%–100% of TERT signal resides outside the nucleus and all other percentages for the class of intermediate localization (I). For each of the 3 classes we determined the number of γH2A.X foci from around 40–100 cells per cell line.

### Analysis of Apoptosis

Hela, U87 and MRC/SV40 cells were transfected with nuclear and mitochondrial TERT shooter. After 2 days they were treated either with 400 µM H_2_O_2_ or 20 Gy irradiation and left for 1 more day (U87 and MRC/SV40) or 2 days for HeLa due to a known delay in apoptosis induction in these cells. After fixation cells were stained with myc-tag for TERT and activated caspase 3 (Abcam) in order to label apoptotic cells. Results have been determined from 30–150 transfected cells per cell line and condition.

### Statistics

One way ANOVA was performed using Sigma Plot (Systat Software Inc, USA).

## Supporting Information

Figure S1 **Immuno-blot of nuclear and mitochondrial fraction in Hela and MCF7 cells treated with 400 **µ**M H_2_O_2_ for 5 days. A:** Immuno-blots from nuclear and mitochondrial fraction in MCF7 and HeLa cells and the indicated days (d0–d5) after H_2_O_2_ treatment as well as untreated cells. Samples for d0 have been taken 3 h after the onset of the H_2_O_2_ treatment. HDAC was used as loading control for the nuclear fraction while coxI was used as loading control for mitochondria. In addition, HDAC staining was performed on mitochondria in order to confirm purity. The detailed method is described in Supporting information methods S1. **B:** Quantification of the ratio between mitochondrial and nuclear TERT over the period of 5 days after H_2_O_2_ treatment.
(TIF)

Figure S2 **TERT intensities in different sub-cellular locations. A:** Representative images showing single TERT immuno-fluorescence staining (red) of HeLa, MCF7 and MRC-5/hTERT cells with and without H_2_O_2_ treatment confirming that the intensive γH2A.X signals in cells with high amounts of DNA damage was not interfering with the nuclear localization signal for TERT. **B:** TERT signal intensity was measured in HeLa, MCF7 and MRC-5/hTERT cells that had been analyzed for the correlation between TERT localization and DNA damage levels and is described in supporting information method S2. The bars are mean and S.E. from 35–100 cells per cell line and condition. *p<0.05.
(TIF)

Figure S3 **Mitochondrial TERT correlates to less DNA damage foci after X-irradiation. A:** MCF7 transfected with nuclear TERT (green) after irradiation with 5 Gy and staining against 53BP1 (red). **B:** MCF7 transfected with mitochondrial TERT (green) after irradiation with 5 Gy and staining against 53BP1 (red). The method is described in supporting information method S3.
(TIF)

Figure S4 **Mitochondrial membrane potential is higher in cells with mitochondrial TERT. A:** HeLa cells transfected with mitochondrial or nuclear TERT before (left) and after irradiation with 20 Gy (right). **B:** MCF7 cells transfected with mitochondrial or nuclear TERT before (left) and after irradiation with 20 Gy (right). The bars indicate means and S.E. * P<0.05, **P<0.01, ***P<0.001. The method is described in supporting information method S4.
(TIF)

Figure S5 **Specificity of the anti-TERT antibody from Rockland. A**: Representative images of TERT immunofluorescence staining using Rockland anti-TERT antibody (ab). Upper row: MRC-5/hTERT cells stained with TERT ab and Alexafluor ^594^ (red) secondary ab (right), while left panel shows DAPI nuclear staining only. Middle row: MRC-5/hTERT cells only stained with secondary antibody. Lower row: MRC-5/hTERT cells stained with same anti-TERT and secondary ab as above, DAPI and TERT signal are merged (left). The right image shows the same staining on MRC-5 cells which are negative for TERT and do not display any staining signal. **B**: Immuno-blot showing a specific band at 127kD for TERT using TERT antibody (Rockland) and a loading control with tubulin. Lane 1: MRC-5/hTERT, 2: MRC-5, 3: HeLa, 4: MCF7. The method is described in supporting information method S5.
(TIF)

Methods S1 **The supporting methods refer to the methods used in Figures S1–S5.** Method S1. Cell fractionation and immune-blotting for the measurement of TERT exclusion after H_2_O_2_ treatment. Method S2. TERT signal intensities for endogenous TERT. Method S3. 53BP1 immuno-staining after X-irradiation in MCF7 cells. Method S4. Measurement of mitochondrial membrane potential after TERT shooter transfection in HeLa and MCF7 cells. Method S5. Immuno-blot for anti-TERT (Rockland).
(DOCX)
