# Supplementary figures and images for: Mitochondrial Telomerase Protects Cancer Cells from Nuclear DNA Damage and Apoptosis
Source: PLoS One. 2013 Jan 9;8(1):e52989. doi: 10.1371/journal.pone.0052989 (PMC3541395; doi:10.1371/journal.pone.0052989)

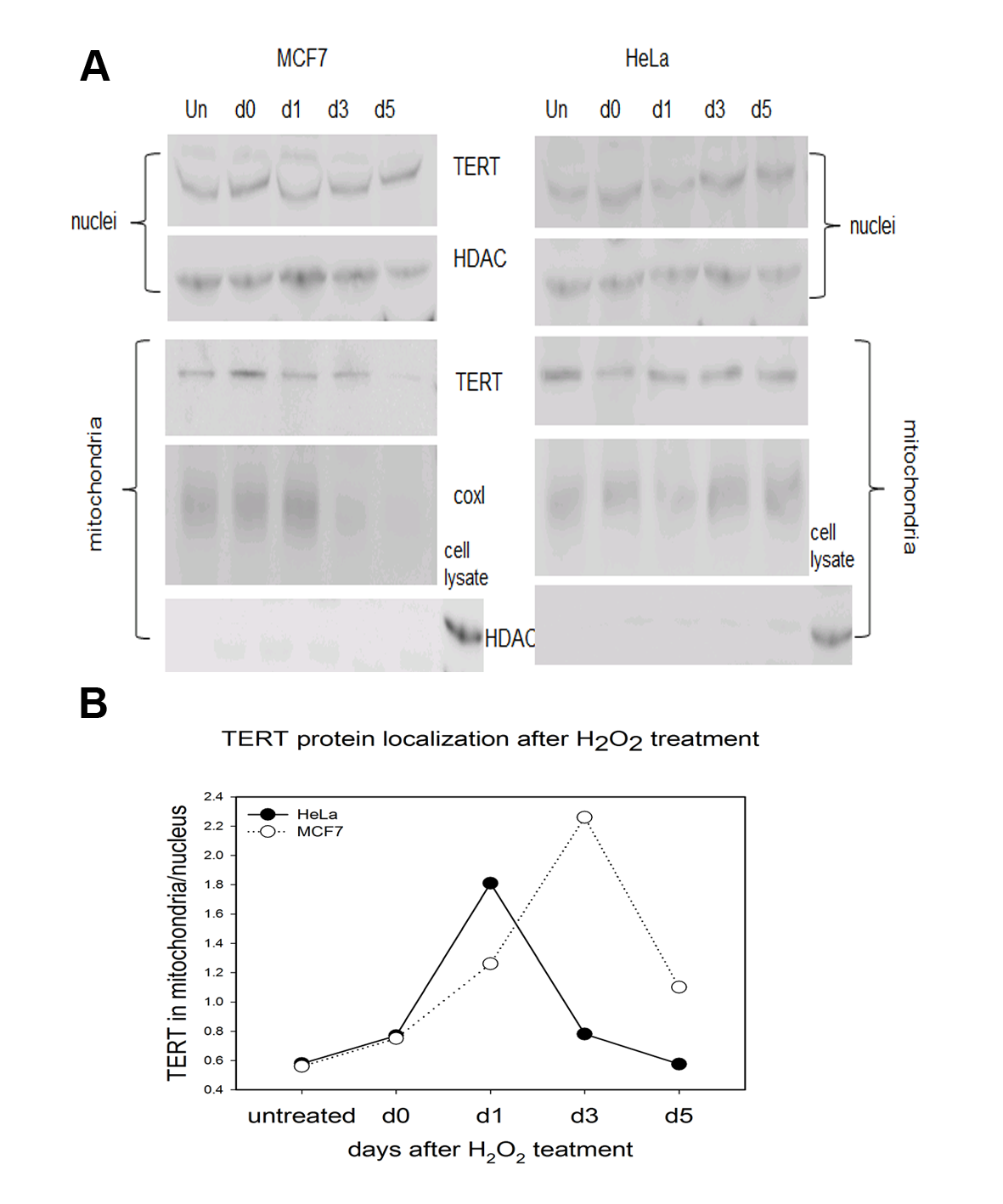

Supplement: Figure S1 — Immuno-blot of nuclear and mitochondrial fraction in Hela and MCF7 cells treated with 400 µM H2O2 for 5 days. A: Immuno-blots from nuclear and mitochondrial fraction in MCF7 and HeLa cells and the indicated days (d0–d5) after H2O2 treatment as well as untreated cells. Samples for d0 have been taken 3 h after the onset of the H2O2 treatment. HDAC was used as loading control for the nuclear fraction while coxI was used as loading control for mitochondria. In addition, HDAC staining was performed on mitochondria in order to confirm purity. The detailed method is described in Supporting information methods S1. B: Quantification of the ratio between mitochondrial and nuclear TERT over the period of 5 days after H2O2 treatment. (TIF) [file pone.0052989.s001.tif]

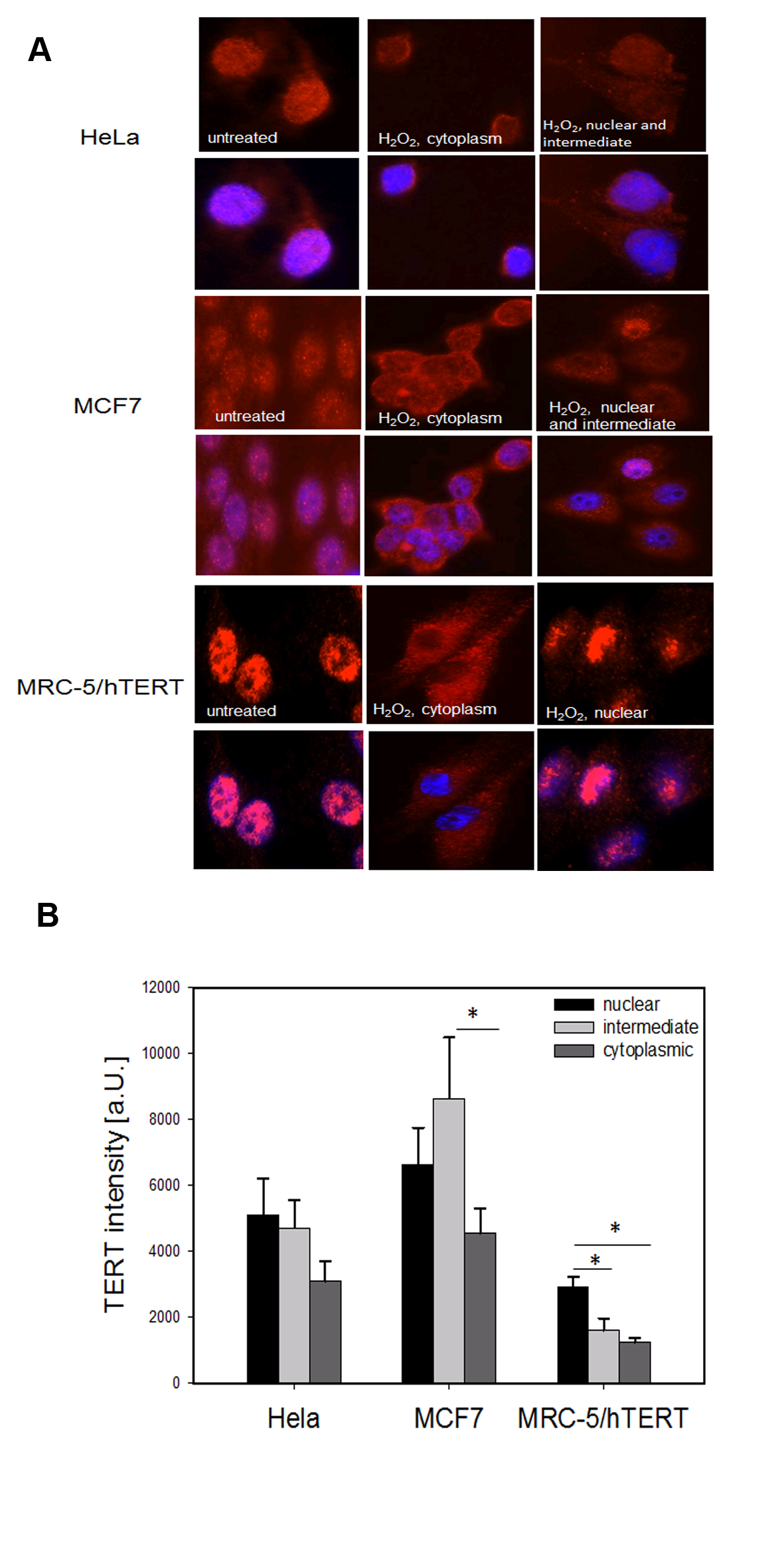

Supplement: Figure S2 — TERT intensities in different sub-cellular locations. A: Representative images showing single TERT immuno-fluorescence staining (red) of HeLa, MCF7 and MRC-5/hTERT cells with and without H2O2 treatment confirming that the intensive γH2A.X signals in cells with high amounts of DNA damage was not interfering with the nuclear localization signal for TERT. B: TERT signal intensity was measured in HeLa, MCF7 and MRC-5/hTERT cells that had been analyzed for the correlation between TERT localization and DNA damage levels and is described in supporting information method S2. The bars are mean and S.E. from 35–100 cells per cell line and condition. *p<0.05. (TIF) [file pone.0052989.s002.tif]

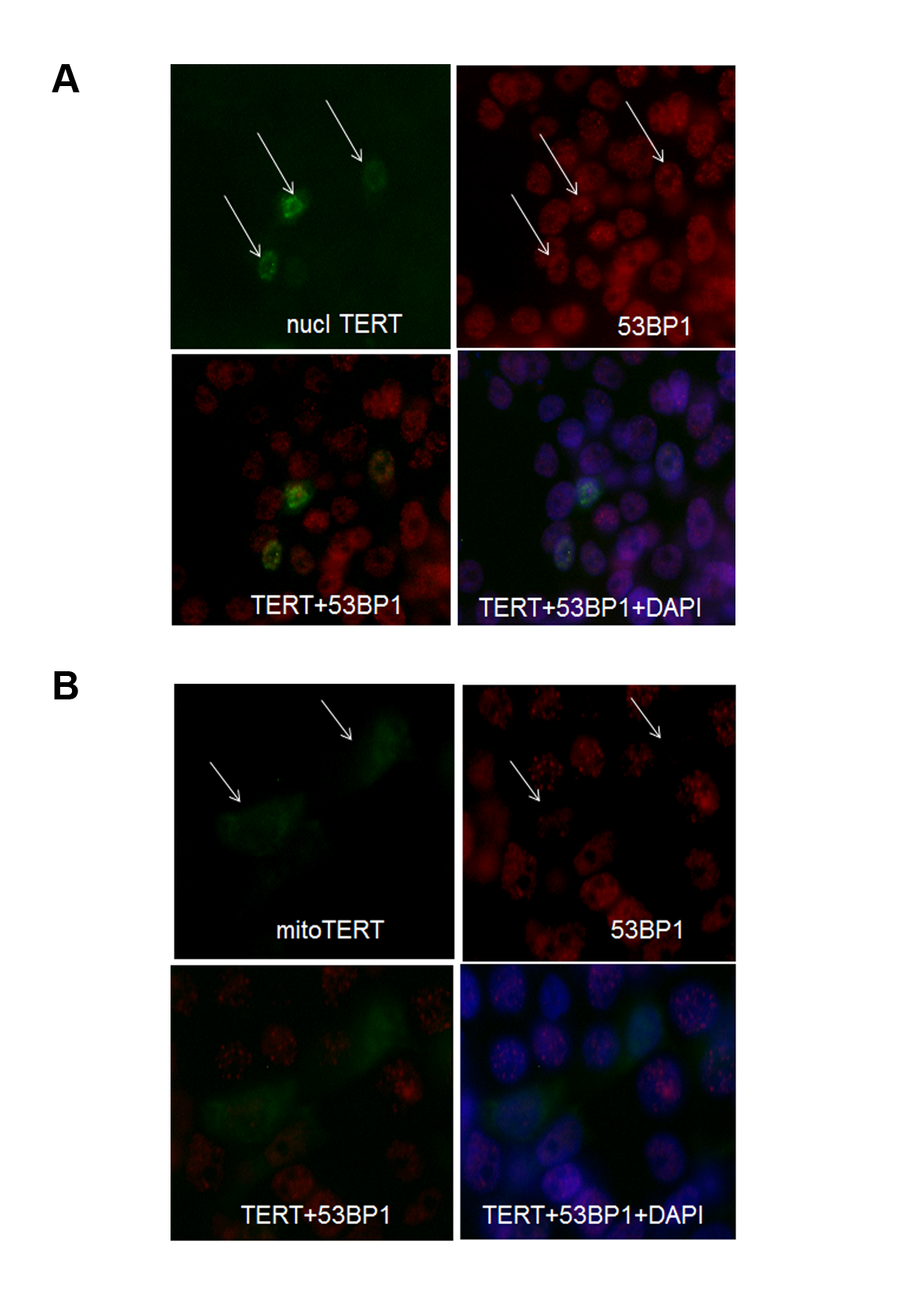

Supplement: Figure S3 — Mitochondrial TERT correlates to less DNA damage foci after X-irradiation. A: MCF7 transfected with nuclear TERT (green) after irradiation with 5 Gy and staining against 53BP1 (red). B: MCF7 transfected with mitochondrial TERT (green) after irradiation with 5 Gy and staining against 53BP1 (red). The method is described in supporting information method S3. (TIF) [file pone.0052989.s003.tif]

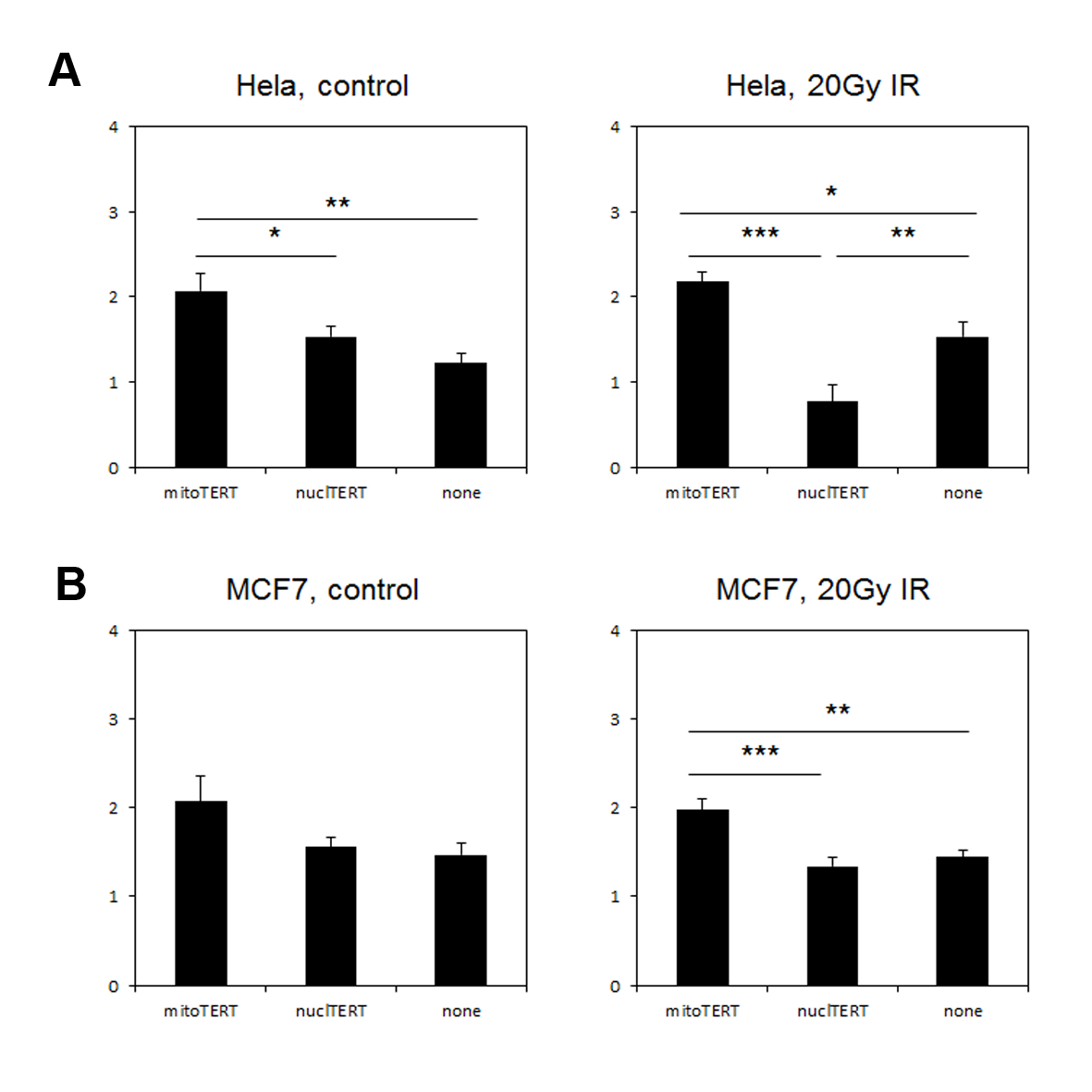

Supplement: Figure S4 — Mitochondrial membrane potential is higher in cells with mitochondrial TERT. A: HeLa cells transfected with mitochondrial or nuclear TERT before (left) and after irradiation with 20 Gy (right). B: MCF7 cells transfected with mitochondrial or nuclear TERT before (left) and after irradiation with 20 Gy (right). The bars indicate means and S.E. * P<0.05, **P<0.01, ***P<0.001. The method is described in supporting information method S4. (TIF) [file pone.0052989.s004.tif]

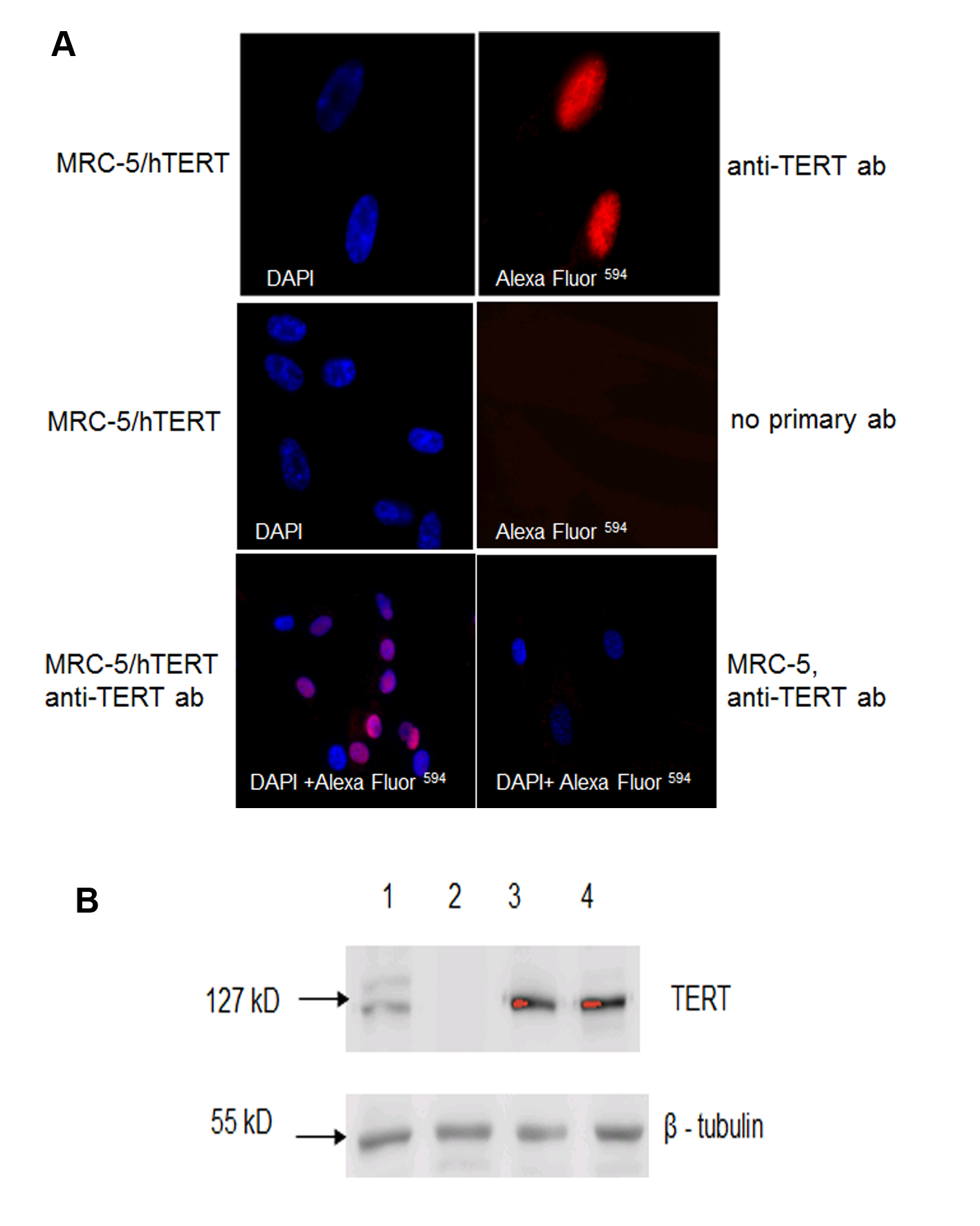

Supplement: Figure S5 — Specificity of the anti-TERT antibody from Rockland. A: Representative images of TERT immunofluorescence staining using Rockland anti-TERT antibody (ab). Upper row: MRC-5/hTERT cells stained with TERT ab and Alexafluor 594 (red) secondary ab (right), while left panel shows DAPI nuclear staining only. Middle row: MRC-5/hTERT cells only stained with secondary antibody. Lower row: MRC-5/hTERT cells stained with same anti-TERT and secondary ab as above, DAPI and TERT signal are merged (left). The right image shows the same staining on MRC-5 cells which are negative for TERT and do not display any staining signal. B: Immuno-blot showing a specific band at 127kD for TERT using TERT antibody (Rockland) and a loading control with tubulin. Lane 1: MRC-5/hTERT, 2: MRC-5, 3: HeLa, 4: MCF7. The method is described in supporting information method S5. (TIF) [file pone.0052989.s005.tif]
