# Supplementary material for: Mitochondrial Telomerase Protects Cancer Cells from Nuclear DNA Damage and Apoptosis
Source: PLoS One. 2013 Jan 9;8(1):e52989. doi: 10.1371/journal.pone.0052989 (PMC3541395; doi:10.1371/journal.pone.0052989)
Supplement: Methods S1 — The supporting methods refer to the methods used in Figures S1–S5. Method S1. Cell fractionation and immune-blotting for the measurement of TERT exclusion after H2O2 treatment. Method S2. TERT signal intensities for endogenous TERT. Method S3. 53BP1 immuno-staining after X-irradiation in MCF7 cells. Method S4. Measurement of mitochondrial membrane potential after TERT shooter transfection in HeLa and MCF7 cells. Method S5. Immuno-blot for anti-TERT (Rockland). (DOCX) [file pone.0052989.s006.docx]

**Supporting Information Methods:**

**Method S1: Cell fractionation and immune-blotting for the measurement of TERT exclusion after H_2_O_2_ treatment**

Fractionation of nuclei and mitochondria used for the results in Fig. S1A on HeLa and MCF7 cells was performed after treatment with 400 μM H_2_O_2_ for 3hours using differential centrifugation as described previously [10].

For the immuno-blots shown and analyzed in Fig. S1A 80 μg nuclear protein and 150 μg mitochondrial protein was loaded onto a 10% PAA gel. For this blot we used a TERT antibody from Epitomics 1:500, HDAC (Abcam) 1:800, coxI (Abcam) 1:500 and HRP-labelled anti-rabbit (Abcam) 1:2000 as secondary antibody.

For the analysis, shown in Fig. S1B the nuclear TERT signal was expressed as the ratio to the HDAC signal, while the mitochondrial TERT signal was expressed as the ratio to coxI. Then the signal of mitochondrial TERT to nuclear TERT was determined and plotted for each time point.

**Method S2: TERT signal intensities for endogenous TERT**

Determination of TERT signal intensities after immuno-fluorescence staining shown if Fig. S2B was performed using ImageJ using the absolute intensity values for the green TERT signal. 35-100 cells were analyzed for each group and cell line.

**Method S3: 53BP1 immuno-staining after X-irradiation in MCF7 cells**

Immuno-staining for 53BP1 as shown in Fig. S3 was performed using a 53BP1 antibody (Cell Signalling) in a 1:100 dilution and a secondary Alexafluor^594^ antibody (Invitrogen).

**Method S4: Measurement of mitochondrial membrane potential after TERT shooter transfection in HeLa and MCF7 cells**

Determination of mitochondrial membrane potential shown in Fig. S4: HeLa and MCF7 cells were seeded on labeled coverslips in order to provide an orientation, transfected with nuclear and mitochondrial TERT. After 2 days the cells were either treated with 200 μM H_2_O_2_ for 1 h or 20 Gy X-irradiation and then stained with 70ng/μl mitotracker green (MTG, Invitrogen) and 16.7nM TMRM (Tetramethylrhodamine, methyl ester, Invitrogen) in serum free medium for 30 min at 37^o^C. After that the coverslips were mounted in PBS and imaged for red TMRM and green MTG fluorescence within 30 min. The conditions were identical for all analyzed samples. Afterwards the coverslips were fixed and the cells stained against myc-tag for TERT and a far red (Alexa-fluor^647^) secondary antibody as well as DAPI for nuclear stain. Afterwards the cells were imaged again and TERT transfected cells identified. For those cells the unfixed images were then measured as a ratio between TMRM and MTG signals.

**Method S5: Immuno-blot for anti-TERT (Rockland)**

For immuno-blots shown in Fig S5B: 80 μg protein (cell lysate) was loaded on a 10% PAA gel. TERT antibody (Rockland, USA) was used 1:500, beta-tubulin (Abcam) 1:1000. The secondary antibody was HRP-labelled anti-rabbit (Abcam) 1:2000.
